# Serum Uric Acid in the PAMELA Study: Main Findings and Association with the Atherogenic Index of Plasma

**DOI:** 10.3390/metabo15100671

**Published:** 2025-10-14

**Authors:** Alessandro Maloberti, Rita Facchetti, Cesare Cuspidi, Guido Grassi

**Affiliations:** 1 Department of Medicine and Surgery, University of Milano-Bicocca, 20126 Milan, Italy; alessandro.maloberti@unimib.it (A.M.); cesare.cuspidi@unimib.it (C.C.); 2 Cardiology 4, “A. De Gasperis” Department, ASST GOM Niguarda, 20159 Milan, Italy

**Keywords:** uric acid, hyperuricemia, atherogenic index of plasma, lipids, cardiovascular risk

## Abstract

Serum uric acid (SUA) overproduction, leading to hyperuricemia, represents a metabolic dysfunction of frequent detection in a number of diseases characterized by an elevated cardiovascular risk, such as metabolic syndrome, essential hypertension, dyslipidemia, obesity, and diabetes mellitus. Similar findings have been also reported for the Atherogenic Index of Plasma (AIP), i.e., a biomarker derived from the logarithmic transformation of the ratio between plasma triglycerides and high-density plasma lipoprotein cholesterol. Both SUA and AIP have been found to be sensitive predictors of fatal and non-fatal cardiovascular events and all-cause mortality, their association representing a highly sensitive marker potentiating the predictive value of each single factor. Although a number of studies have investigated the relationships between SUA and AIP, the association between these two metabolic variables still remains in several indistinct aspects. The present paper, after briefly summarizing the main features of the Pressioni Arteriose Monitorate E Loro Associazioni (PAMELA) study, will review the main study results related to SUA as cardiovascular risk factors. It will also report the original data collected in the PAMELA study on (1) the association between SUA and AIP and (2) the relationships between AIP and normal and elevated blood pressure, metabolic profile, and target organ damage associated with hypertension.

## 1. Introduction

Serum uric acid (SUA) is the purine metabolism final product of both exogenous and endogenous origin. Given the evidence that SUA is synthetized and excreted (mainly by the kidney) in equal amounts, hyperuricemia may result from both overproduction or underexcretion, or a combination of the two mechanisms [1]. Although the most frequent pathological consequences of hyperuricemia are represented by articular gout and renal disease, this metabolic variable has also been associated with cardiovascular (CV) diseases and specifically with chronic coronary syndrome [2,3]. This latter association aroused the interest of the cardiological community, which identified SUA as a biomarker with a major impact on CV risk [2,3].

Regarding the mechanisms leading to hyperuricemia, a decrease in the renal excretion of uric acid is the main determinant of its underexcretion, while a diet rich in purine/fructose, a chemotherapy-induced tumor lysis syndrome, and various metabolic disturbances are the potential causes of its overproduction [1]. Indeed, SUA has been related to several manifestations of the metabolic syndrome, such as the development and progression of high blood pressure (BP) [4], dyslipidemia [5], adiposity/obesity [6], hyperglycemia, and diabetes mellitus [7]. With this background in mind, the relationship between SUA and the Atherogenic Index of Plasma (AIP) results are of interest. This index, firstly described at the beginning of the new millennium, represents the logarithmic transformation of the ratio between serum triglycerides and High-Density Lipoprotein (HDL) cholesterol [8]. The logarithmic transformation is needed to correct the skewed distribution of triglycerides. The index has been related to CV events and mortality [9,10], showing a performance better than the one displayed by other lipid variables. This specific peculiarity is likely to depend on the ability of AIP to integrate both atherogenic (triglycerides at the numerator) and protective (HDL at the denominator) lipid components.

Also, AIP has been related to the metabolic syndrome and its components [11], as previously mentioned for SUA, a finding which brings into question whether and to what extent these two variables are linked together by a clinically relevant relationship. This relationship could have a main direction (SUA increases AIP or vice-versa), could be bidirectional, or could be related to a common factor, the SUA-AIP increase being mainly determined by a single metabolic disturbance.

The present paper aims to describe the main features of the association between SUA and AIP in the general population examined in the context of the epidemiological study known as PAMELA (Pressioni Arteriose Monitorate E Loro Associazioni). The analysis of the data collected in the study will allow us to overcome some of the limitations of previous investigations describing the SUA/AIP profile. These limitations concern the evidence that data were (1) frequently collected in selected very small-size populations, such as those with coronary artery disease or with renal transplantation, (2) not always adjusted for anagraphic, hemodynamic, blood pressure (BP), or metabolic confounders, and (3) based on less sensitive SUA cut-off values for defining CV risk.

## 2. The PAMELA Study: An Overview

### 2.1. Study Profile

The PAMELA study was carried out in Monza (Italy) and 3200 residents, age range 25 to 74 years, were randomly selected. Subjects were stratified for gender and age decades, according to the criteria of the World Health Organization Monitoring Diseases project, allowing us to obtain a group representative of the general population [12]. The initial evaluation was carried out between 1990 and 1993, and the participation rate amounted to 64%, collecting data in more than 2000 individuals [12]. The main objective of this first survey was the definition of the 24 h ambulatory and home BP normality values in the general population. This information, crucial for employing out-of-office BP measurements in clinical practice, was unknown when the study was carried out [12]. Subjects were then reassessed (with another completely analogous evaluation) during the follow-up after 10 years (2002/2003, 1412 subjects) and subsequently after additional 15 years (2017/2018, 562 participants) [12]. On the whole, the full study duration amounted to 25 years, making the PAMELA the epidemiological investigation on ambulatory and home BP with the longest follow-up available so far.

All the scheduled medical visits were performed at Saint Gerardo University Hospital of Monza (a city close to Milan, Italy) during the morning of a working day, after an overnight fast and abstinence from alcohol and cigarette smoking for at least 24 h. Approval of the study protocol was obtained by the Ethics Committee of Milano-Bicocca University.

### 2.2. Measured Variables

For each subject, after signing the study informed consent, a full medical history was collected. During the first and second survey, a mercury sphygmomanometer was used to measure office BP values, while an oscillometric device was used in the third one. Office BP was taken three times in the sitting position and the average value was used for the analyses. Oscillometric BP and heart rate readings during the ambulatory 24 h BP monitoring were carried out every 20 min over the 24 h. Subjects had to perform their usual activities during the daytime period with the indication of holding their arm still during the BP readings. Furthermore, participants were asked to go to bed not later than 11.00 p.m. and to wake up not before 07.00 a.m. Waist circumference was measured in the standing position while height and weight were obtained and used to calculate body mass index. Laboratory analyses included SUA, blood glucose, total plasma cholesterol, HDL cholesterol, triglycerides, and creatinine (from which glomerular filtration rate was estimated—eGFR—by Cockcroft–Gault formulae), and Low-Density Lipoprotein (LDL) cholesterol was calculated according to the Friedewald equation.

Furthermore, a complete echocardiographic evaluation was performed in all the three PAMELA surveys also, with left ventricular and left atrium functional- and structural-focused evaluation. From the second survey of the study, arterial structural (intima-media thickness) and functional (arterial stiffness) parameters have also been evaluated. Finally, fatal and non-fatal CV events and all-cause deaths were recorded, giving the opportunity to thoroughly assess the relationships between BP values (office, 24 h and home), hypertension-related target organ damage, biochemical parameters, and CV events.

## 3. Main SUA Findings of the PAMELA Study

### 3.1. SUA and BP

Our first paper published on SUA reporting original data collected in the context of the PAMELA study was focused on the analysis of the relationships between this metabolic variable and office, ambulatory, and home BP values [4]. Results can be summarized as follows. SUA value came out as one of the major predictors, together with age, body mass index, and plasma glucose, of high BP development during the study follow-up. This was the case when office BP values were considered to define new-onset hypertension, but also when out-of-office BP, namely 24 h and home values, were taken into account [4]. These findings allowed us to conclude that SUA levels represent important predictors of future hypertension, independently of how BP values are measured [4].

A further finding was that SUA is frequently elevated in all the different high BP phenotypes, including sustained hypertension, white-coat hypertension, masked hypertension, and orthostatic and drug-resistant hypertension [13]. Greater SUA values were detected in the sustained hypertension patients while intermediate values were found in the white-coat and masked hypertensive phenotypes as compared to the pure normotensive BP state [13]. In contrast, the non-dipping pattern of the 24 h BP profile was not associated with any substantial difference in SUA values when compared to the data collected in individuals with the physiological nighttime BP drop [13].

Not only absolute BP values but also BP variability is significantly linked to the SUA profile. Indeed, in another analysis of the PAMELA data performed by our group, we found that high SUA levels are associated with a decrease in 24 h BP variability [13]. This was detected for different BP variability measurements (24 h standard deviation, daytime standard deviation, first cyclic component, and the residual uncyclic component), which have been demonstrated to carry a significant prognostic value [14].

### 3.2. SUA and High BP-Related Organ Damage

An additional paper from our group, also based on PAMELA data, focused on the mechanisms through which SUA, in conjunction with hemodynamic and non-hemodynamic factors, may trigger the development and progression of cardiac organ damage, which represents the structural cardiovascular alteration of most common detection in hypertensive patients and closely linked to an increased CV risk [3]. Specifically, SUA was found to strongly predict the development of left ventricular hypertrophy, independently of other confounders, in about 1000 patients evaluated during a 10-years follow-up [15]. Results indeed indicate a 26% increased risk of left ventricular hypertrophy for each 1 mg/dL SUA increase [15]. The relationship between SUA and hypertension-related cardiac organ damage appears to be specific for left ventricular hypertrophy. No significant relationship was found in the PAMELA study between SUA and echocardiographic indices of left atrial enlargement [15]. This finding suggests that SUA may trigger a different effect on cardiac morphology, favoring to a greater extent, factors which are involved in determining the hypertrophic process of the myocardial fibers within the cardiac chambers rather than the atrial remodeling.

### 3.3. SUA and Metabolic Alterations

Data collected in the PAMELA study have also allowed us to provide new information on the relationships between SUA and the metabolic disarray. Indeed, in the data collected during the 10-years follow-up of the study (second PAMELA survey) we have shown a significant association between SUA and the increased risk of new-onset impaired fasting plasma glucose and new-onset diabetes mellitus [7]. As far as the obese condition is concerned, this cardiometabolic alteration identified in 14% of the PAMELA population was associated with elevated SUA values [16,17]. However, SUA in the obese subjects was not found to predict the development of new-onset hypertension and the occurrence of subsequent CV events. It could be possible to speculate that obesity-related factors (insulin resistance, glycemic disturbances, endothelial dysfunction, oxidative stress, autonomic impairment, and sleep apnea) might have overshadowed the role of SUA in promoting the above-mentioned outcomes [17].

## 4. SUA, Lipid Profile, and Adiposity Indices

We have previously mentioned the relationship between SUA and plasma lipids reported in a number of studies, with evidence of a significant correlation with plasma triglycerides [5]. As far as adiposity indices are concerned, previous studies focused on the Visceral Adiposity Index (VAI), while in the PAMELA study, the SUA relationships with Cardio-Metabolic Index (CMI) and Lipid Accumulation Product (LAP) were also assessed [5]. In the 1892 subjects analyzed, SUA correlates strongly with triglycerides (but also with all the evaluated lipids values) as well as with VAI, CMI, and LAP (adiposity indices) [5].

**Figure 1:**
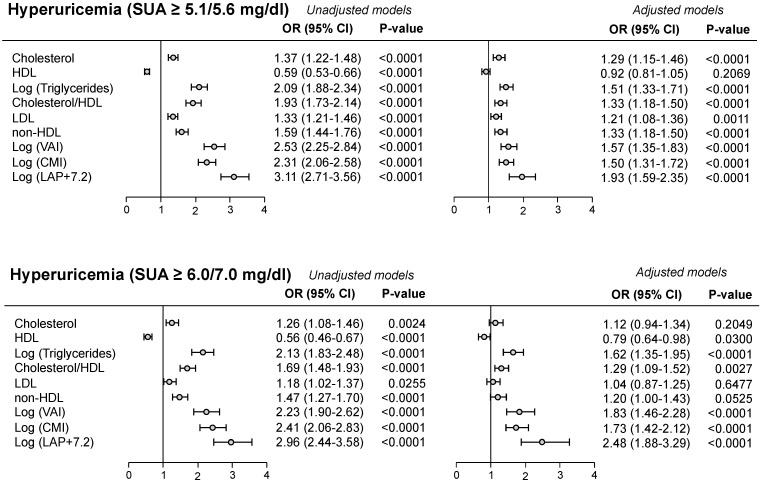
Unadjusted and adjusted logistic model for hyperuricemia (dependent variable) using cut-off values reported in the URRAh study (**upper panel**) and in previous studies (**lower panel**). Adjusted model was included to take into account the number of independent variables in the model itself. Abbreviations: HDL = High-Density Lipoprotein; LDL = Low-Density Lipoprotein; VAI = Visceral Adiposity Index; CMI = Cardio-Metabolic Index; LAP = Lipid Accumulation Product.

**Figure 2:**
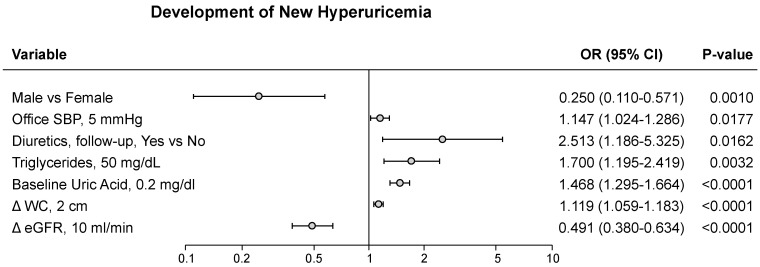
Multiple regression model (linear or logistic) development of new hyperuricemia. Abbreviations: BMI = body mass index; ΔGFR = changes in glomerular filtration rate during follow-up; ΔWC = changes in waist circumference during follow-up; SBP = systolic blood pressure; OR = Odds Ratio. Adapted from Reference [17].

Frequently, the cut-off value for SUA in defining CV risk was the one used in the URic acid Right for heArt health (URRAh) investigation, corresponding to 5.1 mg/dL in females and 5.6 mg/dL in males, because it is more sensitive in predicting future CV events as compared to the classic one (6.0/7.0 mg/dL) [18,19,20]. In the PAMELA study, we found that the cut-off indicated in the URRAh study was significantly related to LDL (Odds Ratio—OR—1.33, *p* < 0.0001) and non-HDL (OR 1.59, *p* < 0.0001, upper panel of Figure 1), the degree of significance being greater than the one detected employing the classic SUA cut-off values [17] (lower panel of Figure 1). This was also the case for the association with the different adiposity indices (Figure 1). Based on these findings, we can suggest that SUA can favor the CV events occurrence at lower cut-offs through pro-atherogenic lipoprotein alterations, while, when SUA levels are markedly increased, also adiposity and general metabolic abnormalities may participate. In the PAMELA study, development of hyperuricemia was associated, at the multivariable analysis, with baseline SUA, home and 24 h BP, female gender, serum triglycerides, and diuretic drug treatment, but also with the increase in waist circumference and the decrease in renal function (Figure 2). These findings therefore suggest that baseline SUA level is one of the most important variables capable of predicting the future development of hyperuricemia.

## 5. Relationships Between SUA and AIP

### 5.1. Results of Published Papers

The relationships between SUA and AIP were assessed in a number of studies, which we identified through a comprehensive literature search of PubMed databases up to 15 August 2025. Only English-written papers were considered. The keywords searching were “atherogenic index of plasma” AND “serum uric acid” with 67 studies retrieved. Fifty-eight papers not focused on the correlation among the two biomarkers were excluded, the 9 remaining papers being discussed thereafter.

In the published studies, SUA showed a significant correlation with AIP [21,22,23,24,25]. However, in most of them, the study population was relatively small (less than 350 subjects) [21,22,23,24] or including very specific subgroups (post-menopausal women [23], patients with coronary artery disease and recent percutaneous revascularization [25], renal transplant recipients [24], and patients with depression [22]). Four studies were carried out in larger population samples. In 645 diabetic patients (53.5% males, age 59.4 ± 12.6 years, mean ± standard deviation) SUA levels were significantly greater in the patient subgroups with higher AIP values to which they were significantly correlated at the univariate analysis (r = 0.154, *p* < 0.001) [26]. Furthermore, results were confirmed with a multivariable model, where AIP is the variable with the highest OR in the relationship with SUA.

In an analysis of the data, collected in 9439 participants enrolled in the NHANES (National Health and Nutrition Examination Survey), a non-linear L-shaped relationship was found between AIP and hyperuricemia prevalence and SUA levels [27]. The AIP saturation point (above which no significant association was detected) amounted to 0.588 for SUA levels and to 0.573 for hyperuricemia, respectively. Below these values, the OR for increased SUA and hyperuricemia was 0.854 (95% CI 0.762–0.946) and 4.4 (95% CI 3.528–5.488), respectively (*p* < 0.001 for both).

In another study, 8258 normal-weight adults with hypertension from the China Hypertension Registry Study were examined [28]. Mean age was 64.9 ± 8.9 (mean ± standard deviation) years and 15.5% of the population suffered from diabetes. Multivariate logistic regression analysis indicated that there was a significant association between AIP and the detection of diabetes mellitus (OR 3.73, 95% CI 2.82–4.94), with an interaction between hyperuricemia and AIP. This means that the association between AIP and diabetes mellitus appears to be more close in hyperuricemic subjects than in those displaying normal SUA.

Finally, in 11,324 subjects (age 53.8 ± 10.6 (mean ± standard deviation) years, 46.3% males, 9.7% with hyperuricemia) from China rural areas, AIP was positively correlated with SUA (r = 0.310 for males, r = 0.347 for females, *p* < 0.001 for both) [29]. When the study population was subdivided into subgroups according to established AIP cut-off values [29], the prevalence of hyperuricemia progressively increased as AIP values were higher. This was confirmed by the multivariate logistic regression analysis where participants in the higher AIP group displayed hyperuricemia (males: OR 2.164; 95% CI 1.782, 2.628; *p* < 0.001; females: OR 2.960, 95% CI 2.311, 3.792; *p* < 0.001). To the best of our knowledge, no longitudinal study has been published so far examining the relationships between SUA and AIP in the long-term period.

### 5.2. Original Data from the PAMELA Study

With this background in mind, we performed an analysis of the PAMELA research project data, examining the behavior of AIP and its relationships with SUA in the general population of the study. The analysis was based on data from 2035 subjects belonging to the first PAMELA survey. Mean age of participants amounted to 50.9 ± 13.7 years and 50.6% of the subjects were males. Mean body mass index was 25.6 ± 4.4 kg/m^2^ and the subjects displayed mean office BP values amounting to 132.9/83.9 ± 21.3/10.6 mmHg. About 20% of the recruited subjects displayed elevated BP values and were under antihypertensive drug treatment. Regarding biochemical variables, average glycemic values were 87.2 ± 13.7 mg/dL, while total cholesterol amounted to 224.1 ± 42.8 mg/dL, HDL cholesterol to 55.5 ± 15.5 mg/dL, and triglycerides to 97.3 ± 16.6 mg/dL. Mean calculated LDL was 145.4 ± 39.1 mg/dL, SUA values were 4.9 ± 1.3 mg/dL, and estimated GFR amounted to 91.8 ± 27 mL/min. All reported values are shown as means ± standard deviation.

**Figure 3:**
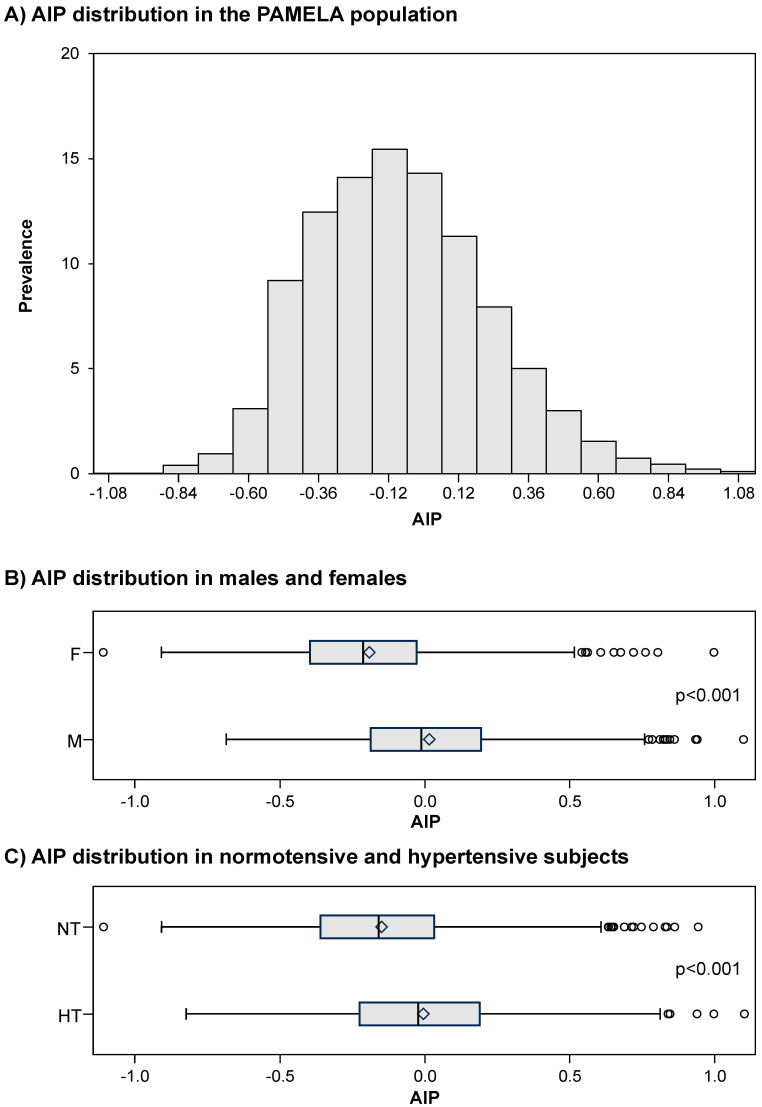
Atherogenic Index of Plasma distribution in the whole PAMELA population (**A**), in males and females (**B**), and in normotensive and hypertensive subjects (**C**). Abbreviations: AIP = Atherogenic Index of Plasma; M = males; F = females; NT = normotensive; HT = hypertensive.

**Figure 4:**
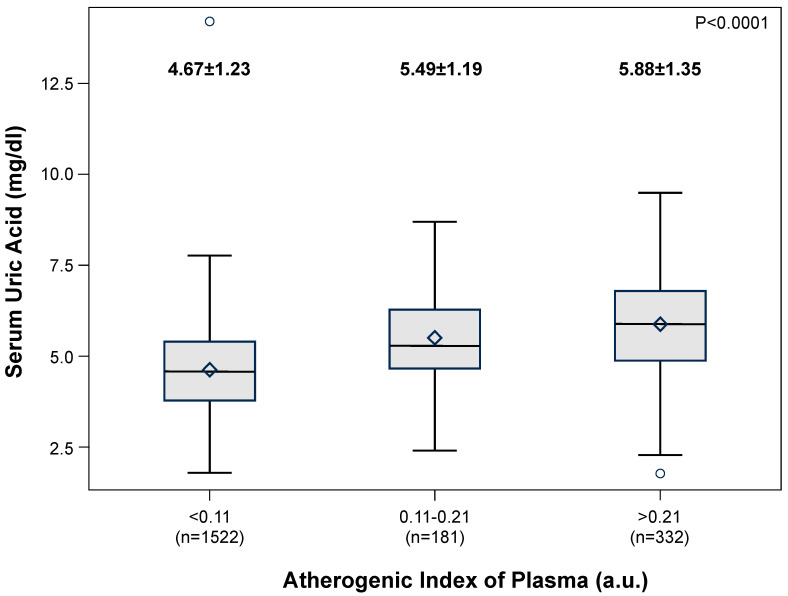
Progressive increase in the Atherogenic Index of Plasma in different patient groups characterized by progressive increases in serum uric acid levels.

**Figure 5:**
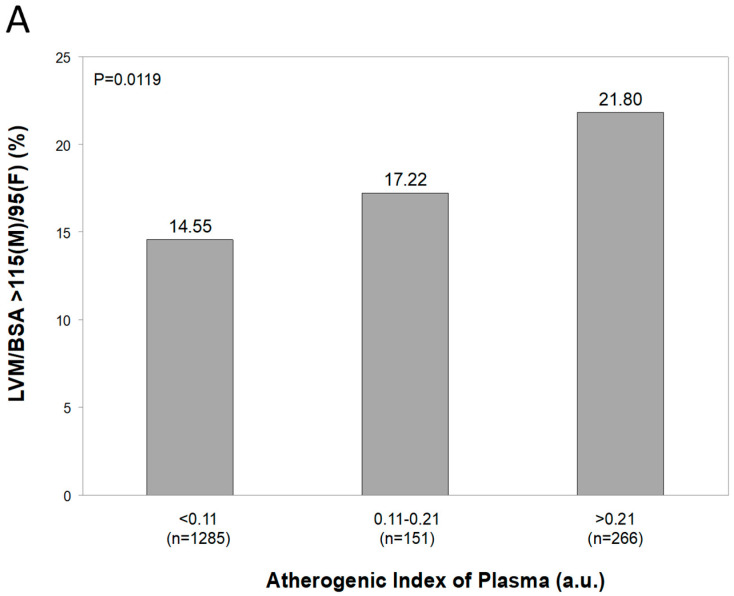

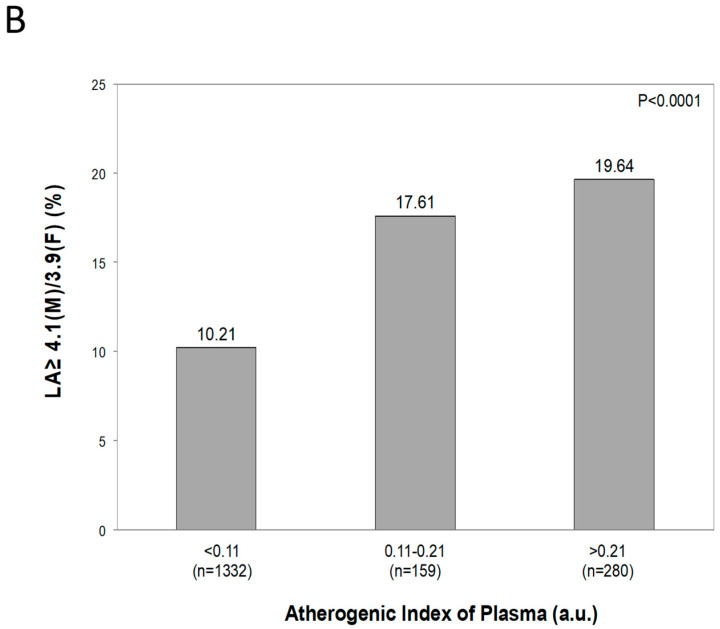
Panel (**A**): Progressive increases in in the Atherogenic Index of Plasma in different patient groups characterized by progressive increases in left ventricular mass index (LVMI) divided by body surface area (BSA). Panel (**B**): Progressive increases in the Atherogenic Index of Plasma in males and female patients characterized by left atrial diameters greater or equal to normal values (4.1 cm and 3.9 cm for males and females, respectively).

Figure 3, panel A, shows the distribution of AIP values in the PAMELA population, the average value amounting to −0.1 ± 0.3 a.u. Male subjects displayed significantly greater AIP values than females (Figure 3, panel B) and this was the case for the hypertensive patients as compared to normotensive controls (Figure 3, panel C). The study population was then subdivided in AIP subgroups as described in a previous study, taking into account epidemiological data showing the relationships with cardiovascular risk [29]: low (AIP < 0.11), intermediate (AIP 0.11–0.21), and high (AIP > 0.21). A progressive, significant increase in SUA values was found from the low to the high AIP subgroups (Figure 4). This was the case also when echocardiographic measurements of left ventricular wall thickness and left atrial dimensions were taken into account (Figure 5, panel A and B, respectively).

As shown in Table 1, by the univariate correlation analysis, all variables considered, excluding heart rate, showed a significant correlation with AIP. Apart from the relationships with HDL cholesterol and triglycerides (which, by definition, are involved in the calculation of AIP values), the strongest correlation was detected for SUA (r = 0.43008, *p* < 0.001). The univariate correlation remained statistically significant in all the subgroups of subjects evaluated (males/females, normotensives/hypertensives, body mass index strata, age below or above 65 years, and use of antihypertensive drugs), except for patients with diabetes mellitus (Table 2). For the multiple linear regression analysis, age, sex, office BP, use of antihypertensive drugs, and total cholesterol and SUA were the variables associated with AIP (Table 3).

## 6. Comments and Conclusions

Original data of the PAMELA investigation reviewed in the present paper strongly support the notion that SUA and AIP are significantly related to each other and that this relationship follows a bidirectional pattern. They also show that AIP is significantly associated with elevated BP values and that this is the case independently of whether BP was assessed in the doctor office, during the 24 h via ambulatory monitoring, or at the patient’s home. Additional findings obtained from the PAMELA data analysis provide unique evidence on the association between metabolic variables, and specifically lipid profile, and AIP. The hypothesis can be thus advanced that AIP may affect BP values via its association with the ipid profile and the related lipid abnormalities.

Two further results of our analysis deserve a brief comment. First, no relationship was found between heart rate and AIP. This finding can be explained by considering that heart rate is the final product of sympathetic/parasympathetic influences on cardiac sinus node activity, largely independent of lipid profile [3]. Second, AIP showed, in the frame of the PAMELA study, a significant association with hypertension-related structural alterations of the cardiac chambers, such as left ventricular hypertrophy and enlargement of the left atrium dimensions. This may suggest that not only hemodynamic factors (such as high BP) but also metabolic alterations affecting lipid profile may participate in the development and progression of cardiac organ damage.

Our data have some limitations. These include the lack of information on the relationships between AIP and vascular structural and functional alterations, such as arterial distensibility and vascular stiffness [3]. They also include the fact that no data are available so far on the relationships between AIP and central aortic BP, which is considered a better predictor of cardiovascular events than peripheral (brachial) BP [3].

In conclusion, the data collected in the frame of the PAMELA study may provide a number of new information on the SUA/AIP relationships with important clinical implications, considering that both these two metabolic variables retain prognostic relevance in different cardiometabolic disease. Future longitudinal studies will allow us to overcome the limitations of the present report and to obtain information on the patterns and consequences of the SUA/AIP relationship during the long-term period.

## Figures and Tables

**Table 1:** Atherogenic Index of Plasma univariate correlation analysis.

Variable	r	*p*-Value
Age	0.24578	<0.0001
Sex (males)	0.34147	<0.0001
BMI	0.34527	<0.0001
SBP Office	0.25679	<0.0001
DBP Office	0.25837	<0.0001
Heart Rate Office	−0.02744	0.2172
Antihypertensive drug	0.18966	<0.0001
Glycemia *	0.29839	<0.0001
Total cholesterol	0.24833	<0.0001
HDL cholesterol	−0.73456	<0.0001
Triglycerides *	0.9382	<0.0001
SUA	0.43008	<0.0001
Creatinine	0.30255	<0.0001
GFR	−0.14117	<0.0001

* log-transformed variables. BMI = body mass index; SBP = systolic blood pressure; DBP = diastolic blood pressure; HDL = High-Density Lipoprotein; SUA = serum uric acid; GFR = glomerular filtration rate.

**Table 2:** Atherogenic Index of Plasma univariate correlation analysis with serum uric acid in subgroup analysis.

Variable	Number	r	*p*-Value
Female	1005	0.39015	<0.0001
Male	1030	0.25628	<0.0001
Age < 65 years	1634	0.43106	<0.0001
Age ≥ 65 years	401	0.35186	<0.0001
No antihypertensive drug	1637	0.42725	<0.0001
Antihypertensive drug	393	0.31914	<0.0001
Office BP < 140/90 mmhg	1174	0.43525	<0.0001
Office BP ≥ 140/90 mmhg	855	0.35746	<0.0001
BMI < 25 kg/m^2^	961	0.34454	<0.0001
25 ≤ BMI < 30 kg/m^2^	757	0.40381	<0.0001
BMI ≥ 30 kg/m^2^	271	0.30076	<0.0001
No diabetes mellitus	1964	0.44639	<0.0001
diabetes mellitus	71	0.06196	0.6078

BMI = body mass index; BP = blood pressure.

**Table 3:** Multiple linear regression model for AIP. Independent variables considered were age, sex, office systolic and diastolic blood pressure, antihypertensive treatment, total cholesterol, creatinine, glomerular filtration rate, and SUA with stepwise selection applied.

Variable	Beta (Std Error)	*p*-Value
Age	0.00048 (0.70585)	0.0009
Sex (Male)	0.01369 (6.21816)	<0.0001
BMI	0.00141 (6.10144)	<0.0001
Antihypertensive drugs	0.01664 (0.27906)	0.0373
Total plasma cholesterol	0.00014 (4.77346)	<0.0001
SUA	0.00545 (4.85739)	<0.0001

_Adj_R^2^ = 0.3019; for symbols and abbreviations, see previous tables.

## Data Availability

The data presented in this study are available on specific request from the corresponding author. The data are not publicly available. The datasets presented in this article are not readily available.
